# A patient with acute hepatitis E and intractable vomiting: a case report

**DOI:** 10.3389/fmed.2025.1614921

**Published:** 2025-07-21

**Authors:** Kui Deng, Xiao-Qin Dong, Dong Xu

**Affiliations:** ^1^Department of Gastroenterology, Yiyang City Central Hospital, Yiyang, Hunan, China; ^2^Department and Institute of Infectious Disease, Tongji Hospital, Tongji Medical College and State Key Laboratory for Diagnosis and Treatment of Severe Zoonotic Infectious Disease, Huazhong University of Science and Technology, Wuhan, Hubei, China

**Keywords:** acute hepatitis E, refractory vomiting, mechanism, treatment, case report

## Abstract

We reported a patient with acute hepatitis E who had recurrent vomiting and dizziness that are uncommon in other patients with hepatitis E. The patient developed dizziness 1 month ago prior, accompanied by yellow urine, sclera, and staining of the skin. Half a month prior, the patient gradually developed fatigue and a poor appetite, accompanied by nausea, aversion to oil, loss of appetite, and itching of the skin. The patient turned out to have an acute hepatitis E infection and was hospitalized for more than a month with repeated vomiting and dizziness. He was treated conservatively with drugs for various reasons. After more than 1 month of medication and rest, the patient's vomiting and dizziness symptoms improved significantly, and follow-up found that the recovery was good.

## Introduction

Acute hepatitis E is a common transmissible disease of the digestive tract and is usually self-limiting. Its onset is similar to that of other forms of acute hepatitis, but it rarely leads to fulminant liver failure or a chronic course of disease in immunocompromised individuals. The incubation period after hepatitis E virus (HEV) exposure ranges from 2 to 6 weeks (1). Infection with HEV usually has a clinically asymptomatic, mild course of disease, and ~5%−30% of infected people will develop acute hepatitis. Symptoms of acute hepatitis include fever, malaise, anorexia, and vomiting, followed by jaundice, brown urine, and liver enlargement. Vomiting as a symptom of acute hepatitis E is reported to mostly occur in the first week after onset (2). Here we reported a patient with acute hepatitis E who had recurrent vomiting and dizziness that are uncommon in other patients with hepatitis E.

## Case presentation

A 66-year-old male presented complaining of dizziness and jaundice for 1 month. The patient also complained of fatigue, poor appetite, and nausea for half a month. The patient developed dizziness 1 month ago prior, accompanied by yellow urine, sclera, and staining of the skin. Half a month prior, the patient gradually developed fatigue and a poor appetite, accompanied by nausea, aversion to oil, loss of appetite, and itching of the skin. On February 20, 2024, the patient was seen at Union Jingshan Hospital of Huazhong University of Science and Technology to check his total bilirubin (TBIL) level of 286.2 μmol/L, alanine transaminase (ALT) level of 1,279 U/L, and aspartate transaminase (AST) level of 2,116 U/L. Anti-HEV IgM was positive (+), and anti-HEV IgG was negative (–). The patient was weakly positive for Epstein–Barr virus IgM (EBV-IgM). Liver magnetic resonance (+), enhancement +Magnetic Resonance Cholangiopancreatography (MRCP) revealed a hemangioma in segment SVI of the liver, multiple cysts of the liver and kidney, and a small amount of fluid around the spleen; MRCP revealed no abnormalities. After treatments to protect the liver and combat infection (Polyene phosphatidylcholine and glutathione), there was no obvious improvement, and jaundice worsened. The patient was transferred to this hospital on February 28, 2024. His previous history included a car accident in 2011, amputation of the right lower limb, previous blood transfusions, and 40 years of drinking, half a kilo/day. The patient's admission diagnosis was acute hepatitis E.

**Figure 1:**
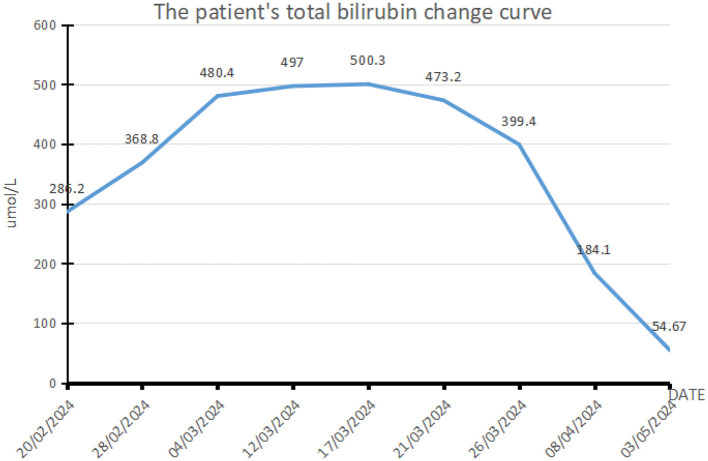
Curve showing changes in the patient's total bilirubin. This patient with acute hepatitis E mainly presents with elevated bilirubin. This figure shows the changes in bilirubin during the patient's treatment. The normal range of total bilirubin is 0–17.1 μmol/L. The maximum bilirubin of the patient increased to 500.3 μmol/L, reaching 29.3 times the reference value (17/03/2024), followed by a drop to 3.2 times (03/05/2024). The patient's vomiting symptoms improved significantly with the decrease of bilirubin.

**Figure 2:**
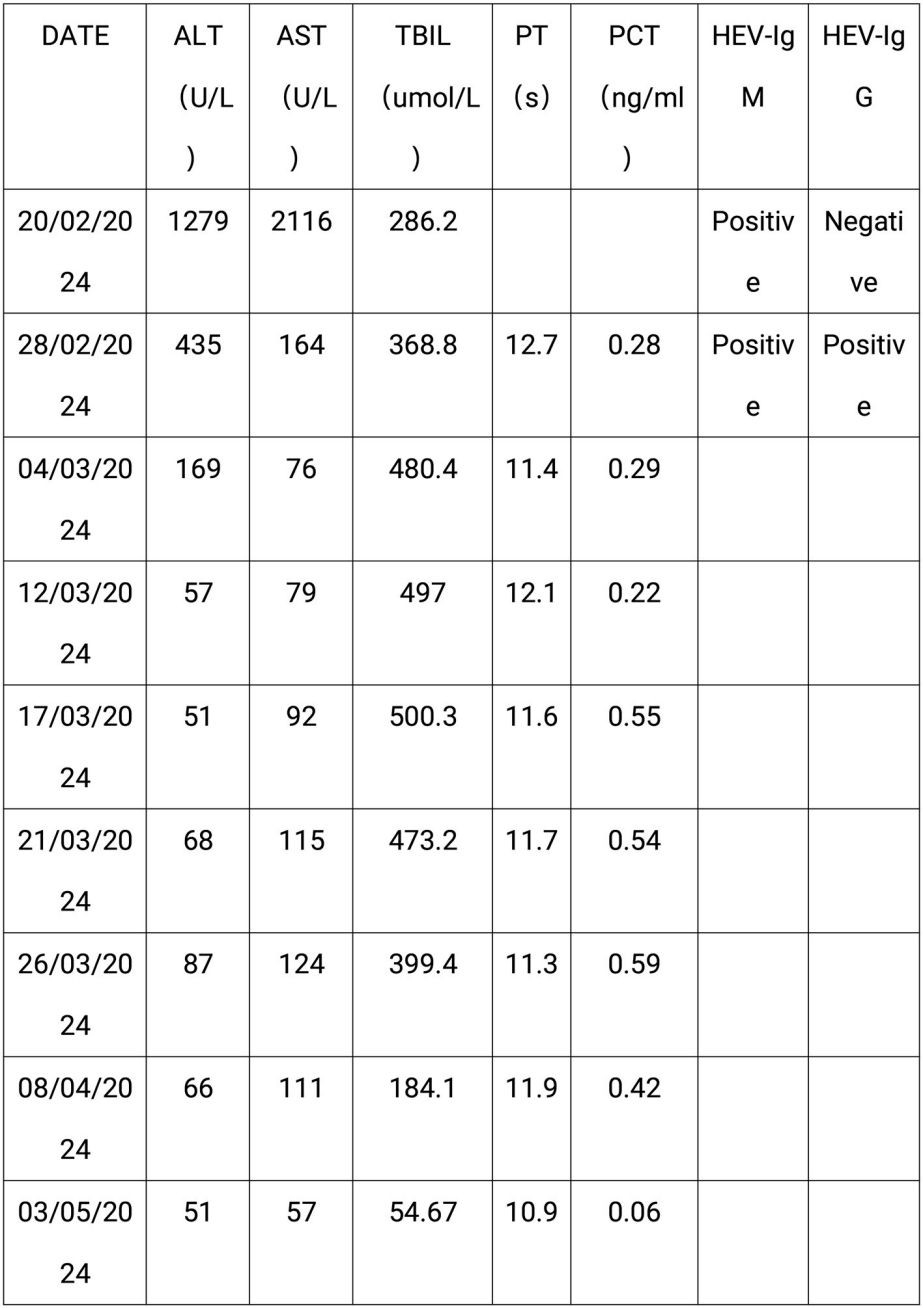
The laboratory test results of the patient. This figure shows the laboratory test results of the patient: AST, aspartate aminotransferase; ALT, alanine aminotransferase; TBIL, Total bilirubin; PT, prothrombin time; PCT, procalcitonin; HEV-IgM, Anti-HEV IgM; HEV-IgG, Anti-HEV IgG.

**Figure 3:**
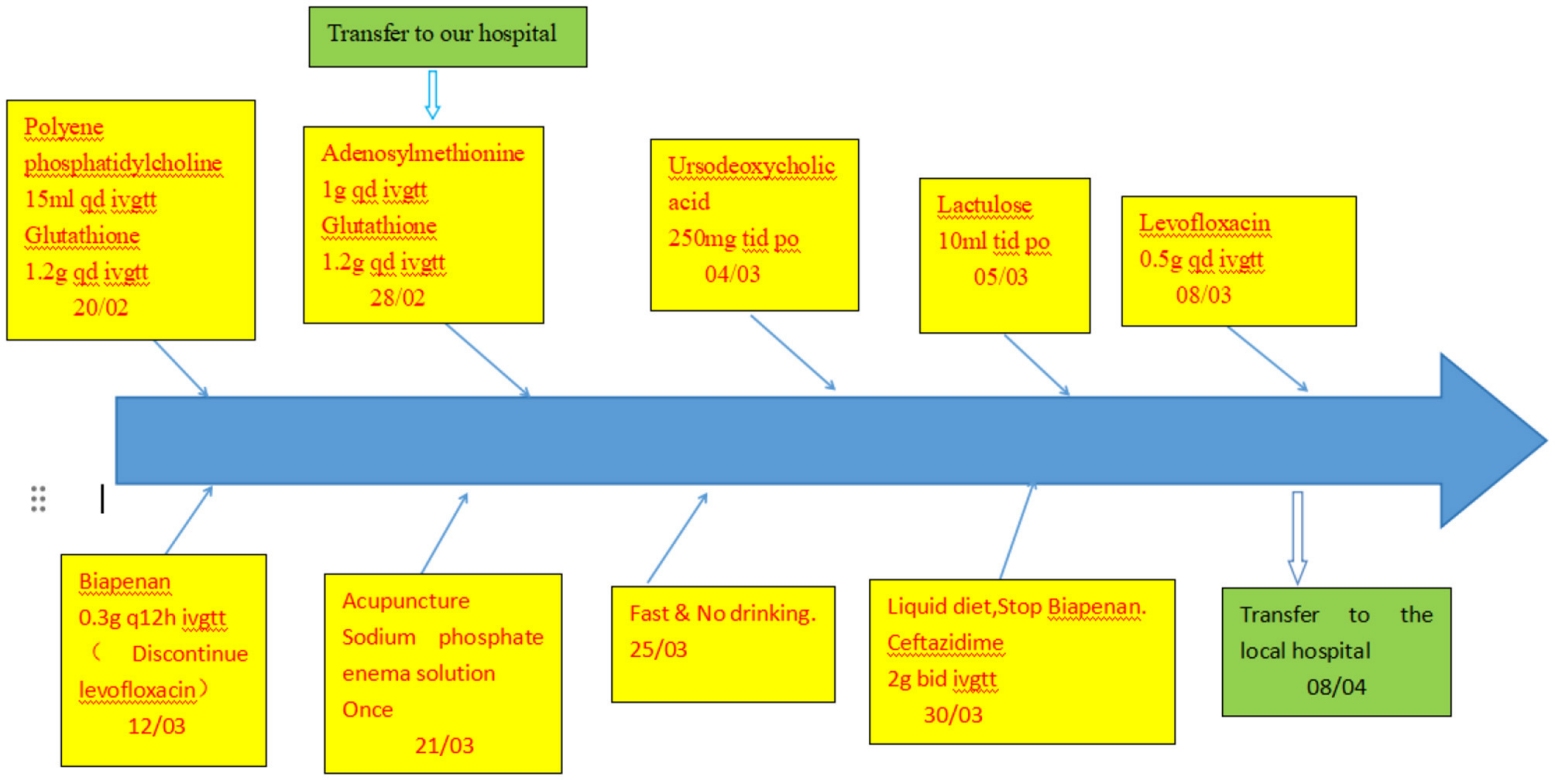
Schematic diagram of the patient's treatment process.

**Figure 4:**
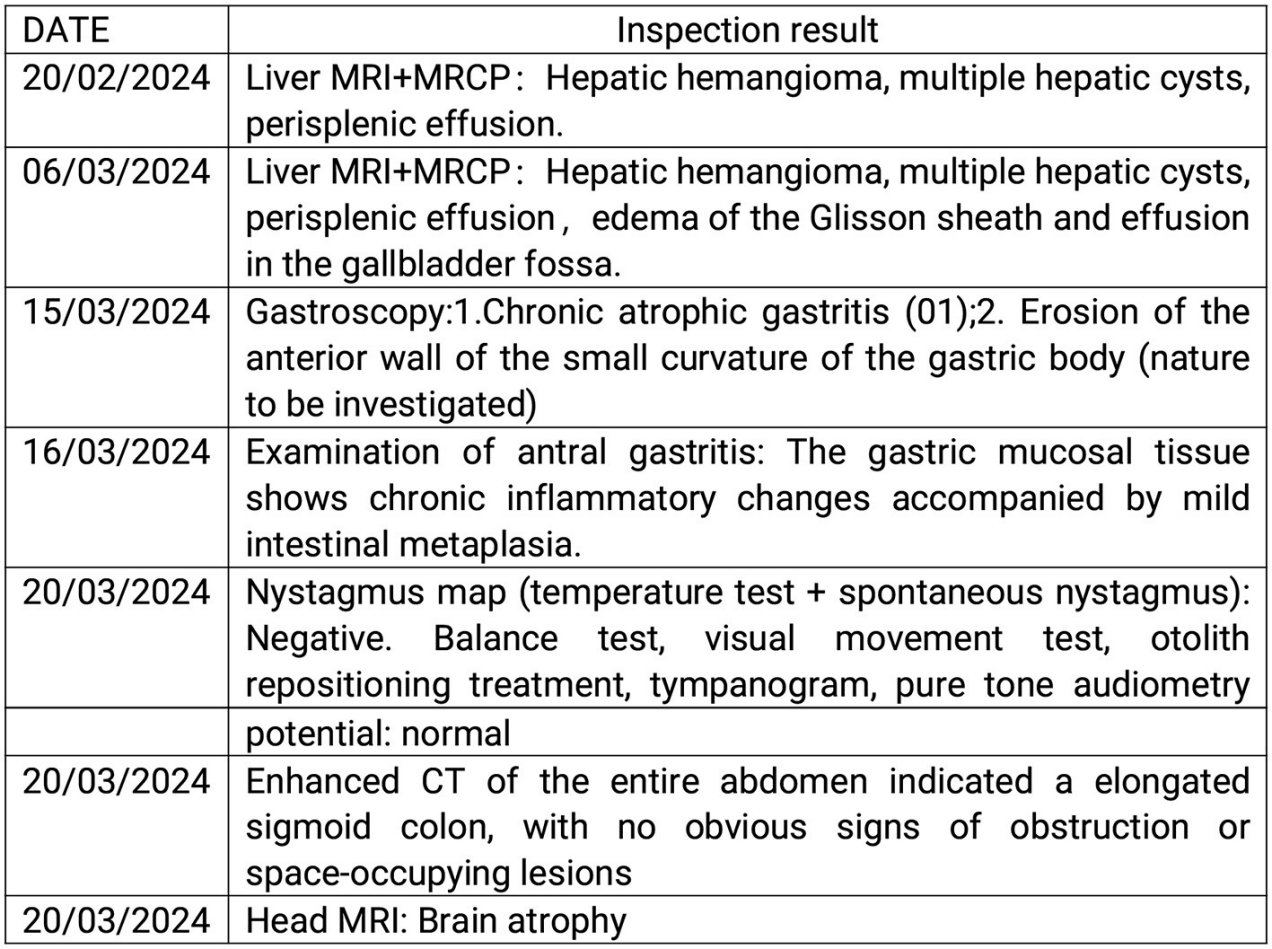
The examination results of the patient. MRI, magnetic resonance imaging; MRCP, magnetic resonance cholangiopancreatography; CT, computed tomography.

**Figure 5:**
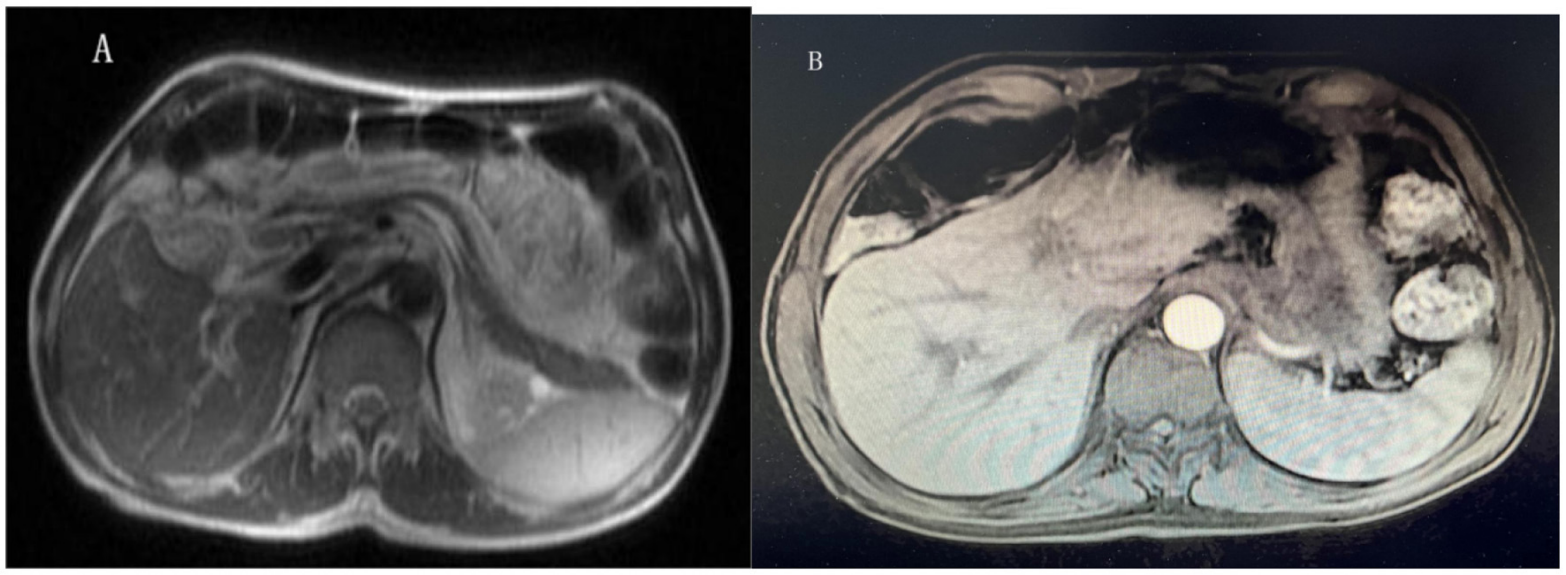
Liver magnetic resonance imaging (MRI). **(A, B)** Showed the hepatic hemangioma, multiple hepatic cysts, perisplenic effusion, edema of the Glisson sheath and effusion in the gallbladder fossa.

**Figure 6:**
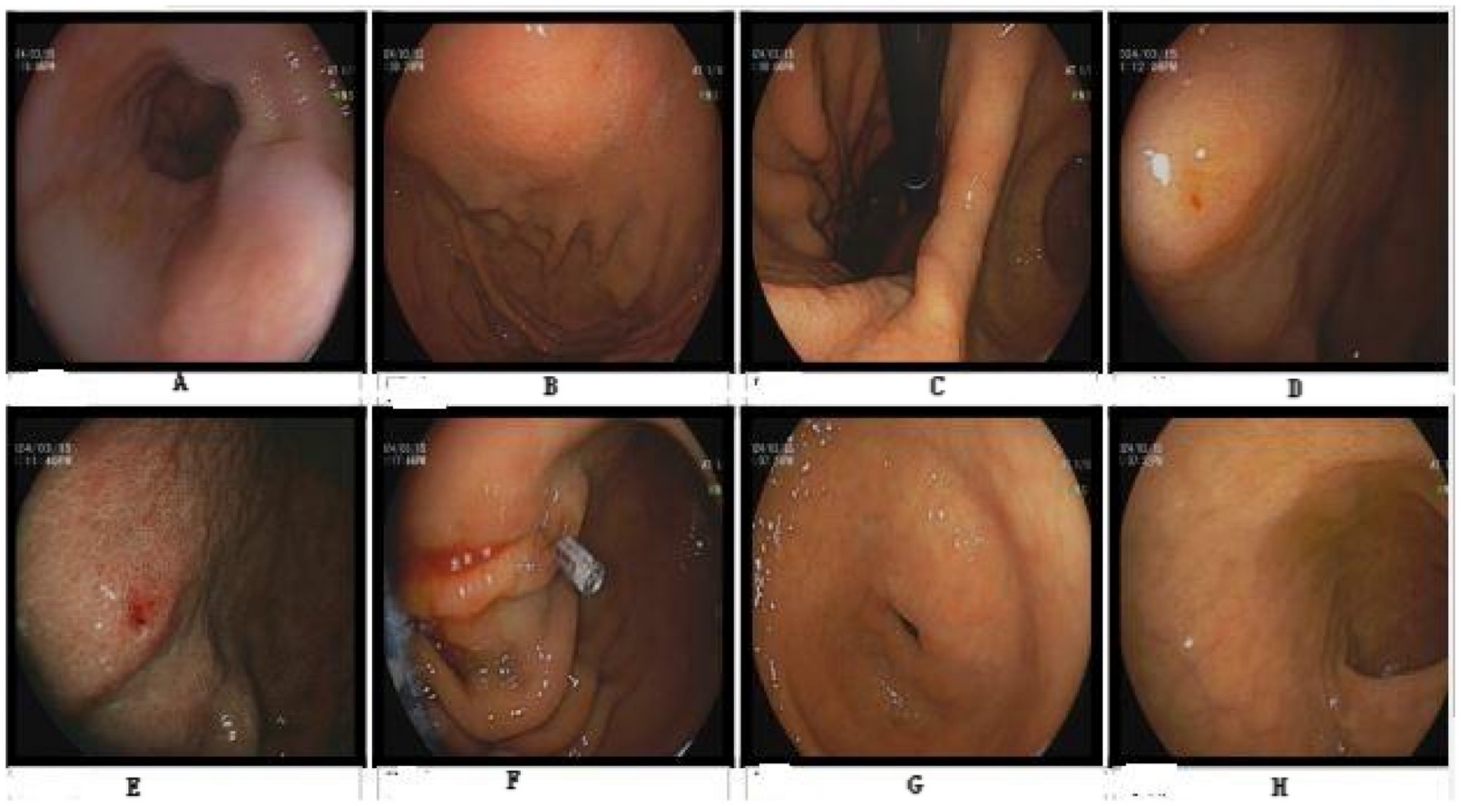
Gastroscopy. No abnormalities were observed in **(A–D, H)**. **(E)** Shows erosion of the gastric body. **(F)** Shows that the titanium clip stops bleeding. **(G)** Shows atrophy of the antrum of the stomach.

**Figure 7:**
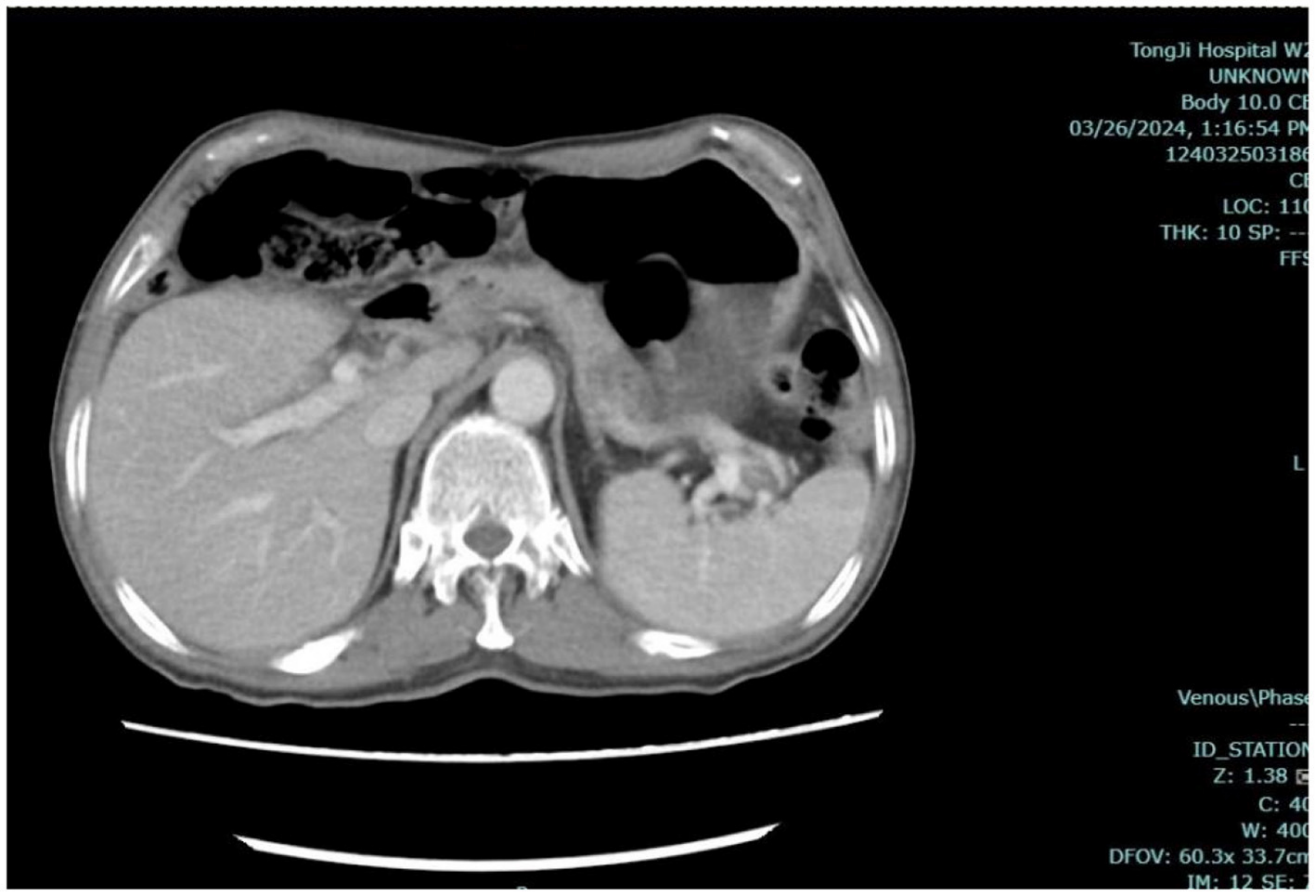
Abdominal CT. Enhanced computed tomography (CT) of the entire abdomen indicated a elongated sigmoid colon, with no obvious signs of obstruction or space-occupying lesions.

**Figure 8:**
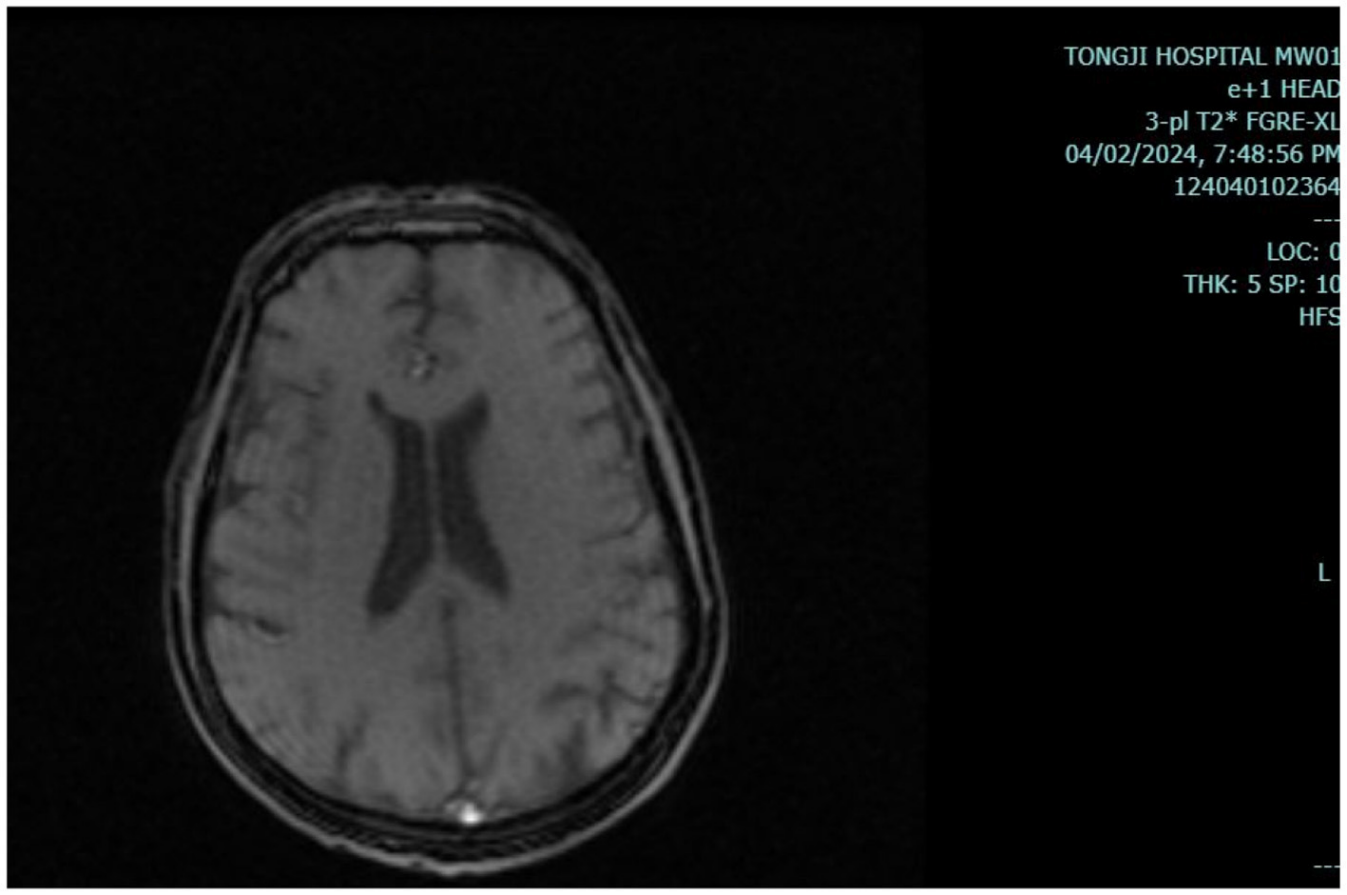
Head MRI. Head magnetic resonance imaging (MRI) shows brain atrophy.

Results of liver function tests on the day of admission were as follows (Figures 1, 2): a TBIL level of 368.7 μmol/L, an ALT level of 435 U/L, and an AST level of 164 U/L. Blood coagulation was normal. Autoantibody profiling revealed an elevated antinuclear antibody titer with a cytoplasmic pattern of 1:100, and an elevated antinuclear antibody titer with a nuclear pattern of 1:3,200. Other viral hepatitis antibodies were negative. EBV-IgM and Cytomegalovirus IgM were also negative. Alpha-fetoprotein and protein induced by vitamin K absence-II were normal. After admission, the patient was given levofloxacin and glutathione treatment to protect the liver (Figure 3). In addition to drug treatment, we supplemented the patient with multivitamins. Liver magnetic resonance imaging (MRI; Figures 4, 5) on March 6 revealed a small cyst in the left outer lobe of the liver and a hemangioma in the right posterior lobe of the liver. Additional findings included: Glisson sheath edema, multiple cysts in both kidneys, effusion in the gallbladder fossa, and slight effusion around the spleen, and the antrum wall and duodenal wall were thickened and edematous. Amylase and lipase levels were normal. The patient still had symptoms of fatigue, vomiting, and itchy skin after those treatments. Hormone therapy was recommended, but the patient's family refused it due to adverse reactions. The patient lacked his right lower limb, so blood vessels of the left lower limb would be selected for artificial liver support, which may lead to disability of both legs if deep venous thrombosis of the lower extremity occurred. Artificial liver support was postponed after consultation with the patient's family. On March 12, the TBIL level was 497 μmol/L and procalcitonin (PCT) level was 0.59 ng/ml. Considering the patient's right upper abdominal pain and the presence of fluid in the gallbladder fossa, levofloxacin treatment was escalated to biapenem to prevent cholecystitis and sepsis (Figure 3). The patient still experienced repeated vomiting, several times a day, that was aggravated after eating, and he also had reduced defecation, which was inconsistent with the common clinical manifestations of hepatitis E. To exclude digestive tract diseases and prepare for hormone therapy, painless electronic gastroscopywas performed on March 15, 2024. Gastroscopy revealed chronic atrophic gastritis (O1) and erosion of the anterior wall of the lesser curvature of the stomach (nature to be determined; Figures 4, 6). After biopsy, control of bleeding was difficult, and a titanium clip was used by the endoscopist to clamp the wound. Examination of the gastric mucosal tissue from the antrum of the stomach revealed chronic inflammatory changes with mild intestinal metaplasia. Given the high risk of gastrointestinal bleeding, hormone therapy was abandoned. Further refinement of the nystagmus map (temperature test + spontaneous nystagmus) was negative. Results of a balance test, visual motor test, otolith reduction, tympanogram, and the pure-tone threshold were normal (Figure 4). A complete enhanced computed tomography (CT) image of the abdomen revealed that the sigmoid was lengthy, though there were no obvious signs of obstruction or space occupied by a legion (Figures 4, 7). Levels of cortisol and adrenalin were normal. An MRI of the head revealed cerebral atrophy (Figure 8). The patient had difficulty tolerating the colonoscopy due to his physical condition, so it was abandoned. To determine further causes of the vomiting, we organized a hospital-wide consultation, which ruled out digestive tract obstruction, tumors, pancreatitis, otoliths, and endocrine and nervous system diseases based on the previous examination. Acupuncture treatment is considered to have therapeutic effects on nausea and vomiting in various disease (3). Therefore, on the suggestion of the Traditional Chinese Medicine Department, we adopted this measure. As of March 21, the patient's bilirubin level tended to decrease (Figure 1), but dizziness still occasionally occurred, and vomiting, itching of the skin, fatigue, and constipation were not relieved, so he was treated with a sodium phosphate enteric enema. As of March 25, defecation had nearly stopped, so fasting and water consumption were recommended (Figure 3). By March 30, the patient's vomiting began to improve, and he resumed a liquid diet. Anti-infective treatment was switched to ceftazidime starting on March 30 (Figure 3). The vomiting subsided until April 03. On April 08, the patient's bilirubin level was 184.1 μmol/L, and the patient was referred back to a local hospital for further treatment. After returning to the local hospital, drug-based treatment to protect the liver was continued. On May 3, the patient's bilirubin level decreased to 54.67 μmol/L.

## Discussion

In the current case, the patient's vomiting lasted for more than a month, which was quite different from that of most other patients with hepatitis E. Examinations can rule out common diseases that cause vomiting, such as upper digestive tract diseases, intestinal obstruction, digestive tract tumors, pancreatic diseases, endocrine diseases, a lesion occupying cranial space, and vestibular dysfunction (4). The symptoms of vomiting and dizziness were relieved after bilirubin decreased, so we believe that these symptoms were related to the hepatitis E infection itself. The cause of vomiting was probably related to viremia, dyspepsia caused by high bilirubin levels, or gastrointestinal dysfunction caused by toxins.

We all know that the secretion of digestive enzymes in patients with acute hepatitis is reduced. Furthermore, cholestasis exists in acute hepatitis, and the latter can lead to a reduction in the secretion of bile acid salts. A deficiency of bile acids can lead to atrophy and morphological changes of the intestinal mucosa, as well as a reduction in electromyophysiological activities (5). On the other hand, when patients have cholestasis, the expression of the Fas gene in the intestinal mucosa may increase. The high expression of this gene can accelerate the apoptosis of B lymphocytes, thereby leading to the damage of the intestinal immune barrier (6). The decline of intestinal function is the basis of weakened intestinal motility. Acute hepatitis E may cause symptoms of indigestion such as nausea and vomiting by reducing the secretion of digestive enzymes and weakening intestinal motility function. Our patient once experienced the symptom of almost complete cessation of exhaust and defecation from the anus, which confirmed the weakened intestinal motility function.

Additionally, HEV infection can cause liver damage, as well as damage to the nervous system, kidneys, blood system, and pancreas; the mechanisms for this are still unclear, but direct damage caused by extrahepatic replication of HEV cannot be ruled out (7). Hepatitis E is transmitted via the digestive track, but whether it replicates in the gastrointestinal tract and directly causes gastrointestinal damage needs to be studied further. Concomitant neurological conditions caused by HEV include facial nerve palsy, Guillain–Barré syndrome, meningitis, myelitis, mononeuritis multiplex, and brachial neuritis (8, 9). Studies have shown that HEV virus can complete the entire viral life cycle in oligodendrocytes and can also penetrate the blood–brain barrier (10–12). HEV can infect the mitochondria of the brain tissue of gerbils and induce apoptosis (10). These studies indicate that HEV may have a certain neurotropism, which may be one of the mechanisms leading to dizziness and central vomiting in the current patient.

The patient was admitted with a high antinuclear antibody titer, but autoimmune liver disease was not considered because acute hepatitis itself can also cause an increase in antinuclear antibodies. Our patient was not at risk for autoimmune liver disease (middle-aged and young people, more women). That said, HEV-IgM was elevated and HEV-IgG changed from negative to positive (13) in this patient. These factors suggest that the current patient be diagnosed with acute hepatitis E.

Most acute HEV infections should be treated conservatively while waiting for spontaneous clearance. There are no specific drugs approved for hepatitis E to date (7). However, short-term use of ribavirin may be considered. At present, a number of studies overseas have used ribavirin to treat acute hepatitis E, and the evaluation index is the degree of decrease in HEV-RNA. However, there is no unified standard for the determination of HEV-RNA to date. Some studies contend that the HEV-RNA values obtained with different methods of detection can vary by 100–1,000-fold (14). The individual dosage varies greatly for ribavirin treatment, and there is no uniform standard (15, 16). Other options, such as hormone therapy and artificial liver support, were not suitable for the current patient, as mentioned earlier. The treatment of hepatitis E in most patients, and especially those with liver failure, takes a long time, and the most important bridges are rest and supportive treatment.

## Conclusion

We described an elderly male patient with acute hepatitis E, with unexplained vomiting and dizziness during the course of the disease. Hyperbilirubinemia is an important cause of repeated vomiting in patients with HEV. HEV may have a certain neurotropism, which may be one of the mechanisms leading to dizziness and central vomiting in the current patient. Rest and supportive care are important in the management of acute hepatitis E. Owing to its increasing incidence, standardized treatment guidelines are urgently needed. images.

## Data Availability

The original contributions presented in the study are included in the article/supplementary material, further inquiries can be directed to the corresponding authors.
